# Risk Assessment of Tuberculosis in Patients With Chronic Mental Illness and Related Factors: A Population‐Based Cohort Study in Taiwan

**DOI:** 10.1111/crj.70088

**Published:** 2025-06-09

**Authors:** Li‐Chen Hung, Pei‐Tseng Kung, Tung‐Han Tsai, Wen‐Chen Tsai, Kuang‐Hua Huang

**Affiliations:** ^1^ Department of Healthcare Management Yuanpei University of Medical Technology Hsinchu Taiwan; ^2^ Department of Healthcare Administration Asia University Taichung Taiwan; ^3^ Department of Medical Research China Medical University Hospital Taichung Taiwan; ^4^ Department of Health Services Administration China Medical University Taichung Taiwan

**Keywords:** epidemiology, mental illness, *Mycobacterium tuberculosis* infection, psychiatric disease, tuberculosis

## Abstract

**Introduction:**

Tuberculosis (TB) is a globally prevalent chronic infectious disease. The World Health Organization estimates that mental illnesses will become the leading cause of global disease burden in 2030. The inability to detect and provide proper treatment for TB in mental illness patients is an epidemic prevention blind spot. The objective of this study was to retrospectively compare the incidence of TB between the general public and mental illness patients.

**Methods:**

This study used data across Taiwan from 2002 to 2013. The National Health Insurance Research Database, Registry for Catastrophic Illness Patients, Tuberculosis Database, and Household Registration Records of Taiwan were analyzed. Propensity score matching was used to reduce basic characteristic differences between mental illness patients and the general public. The conditional Cox proportional hazards model and cumulative risk curve were used to compare their risk of developing TB.

**Results:**

It was shown that TB incidence was 87 and 71 per 100 000 person‐years in mental illness patients and the general public, respectively. The risk of developing TB in mental illness patients was 1.48 times (95% CI: 1.38–1.59) that of the general public.

**Conclusion:**

Mental illness patients are a high‐risk population for TB and should be listed as key subjects for TB prevention and control.

## Graphical abstract

The risk of developing tuberculosis among patients with mental illness is higher than that in the general population, and this risk disparity increases over time. Mental illness should therefore be considered a high‐risk factor for tuberculosis. Implementing tuberculosis screening in this population may aid in the early identification of potential cases.

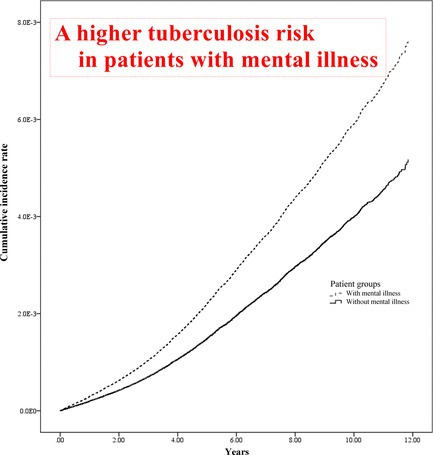

## 1 Introduction

Tuberculosis (TB) remains a globally prevalent and notifiable disease, with delayed diagnosis contributing to community transmission due to its long incubation period and nonspecific symptoms. In 2017, TB affected over 10 million people worldwide, including 5.8 million men, 3.2 million women, and 1 million children1. It impacts all age groups, with 90% of cases occurring in individuals over 15 years old and 9% coinfected with HIV [1]. Risk factors for TB include sex, age, education, income, living conditions, and comorbidities such as diabetes and chronic kidney disease (CKD) [2, 3, 4, 5, 6, 7, 8, 9, 10, 11, 12]. Furthermore, if a person who is infected with Mycobacterium TB has comorbidities, the organism may activate, increasing the probability of developing TB [13]. Thus, the presence of comorbidities should also be considered when examining the risk of developing TB. Interestingly, studies have pointed out that mental illness will increase the risk of developing TB [14, 15, 16, 17].

Mental disorders affect one in four people globally and are projected to be the leading cause of disease burden by 2030 [1]. In Taiwan, their prevalence has risen from 11.5% in 1990 to 23.8% in 2010, underscoring their growing public health impact [18]. Studies across multiple countries show higher TB rates in patients with mental disorders compared to the general population. For example, in Nagasaki, Japan, TB incidence among mental health patients was 2.52%, significantly higher than the general public [2], with elevated risks for inpatients and staff in shared wards [12]. In Russia, severe mental illness patients had a 15% TB infection rate [16]. In the United States, psychiatric hospital patients showed higher tuberculin positivity [6], and nearly 90% of mental health residents in a rehabilitation center exposed to TB patients were infected [9]. A retrospective study in Taiwan also found elevated TB incidence in schizophrenic patients [4].

The above findings suggest that patients with mental illness may have a higher risk of developing TB. Thus, this study used the nationally representative National Health Insurance Research Database (NHIRD) to assess TB incidence in patients with mental illness compared to the general population and explore correlation factors.

## 2 Methods

### 2.1 Data Sources

In this study, data from the Registry for Beneficiaries, the Registry for Catastrophic Illness, the Tuberculosis Database from the Department of Statistics of the Ministry of Health and Welfare of Taiwan, and the Household Registration Records from the Ministry of the Interior of Taiwan were analyzed. The Tuberculosis Database is an open national database from 2002 to 2013 released by the Taiwan Centers for Disease Control. Disease diagnoses in the database are established in accordance with the International Classification of Diseases, Ninth Revision, Clinical Modification (ICD‐9‐CM). The database above is used to obtain real‐world data to support clinical decision‐making and healthcare policymaking [19, 20].

This study was performed in accordance with the Declaration of Helsinki. This study involving humans was approved by the Institutional Review Board in Taiwan. Participant consent was not required because this was a retrospective study using deidentified data; the need for informed consent was waived.

### 2.2 Study Subjects

**FIGURE 1:**
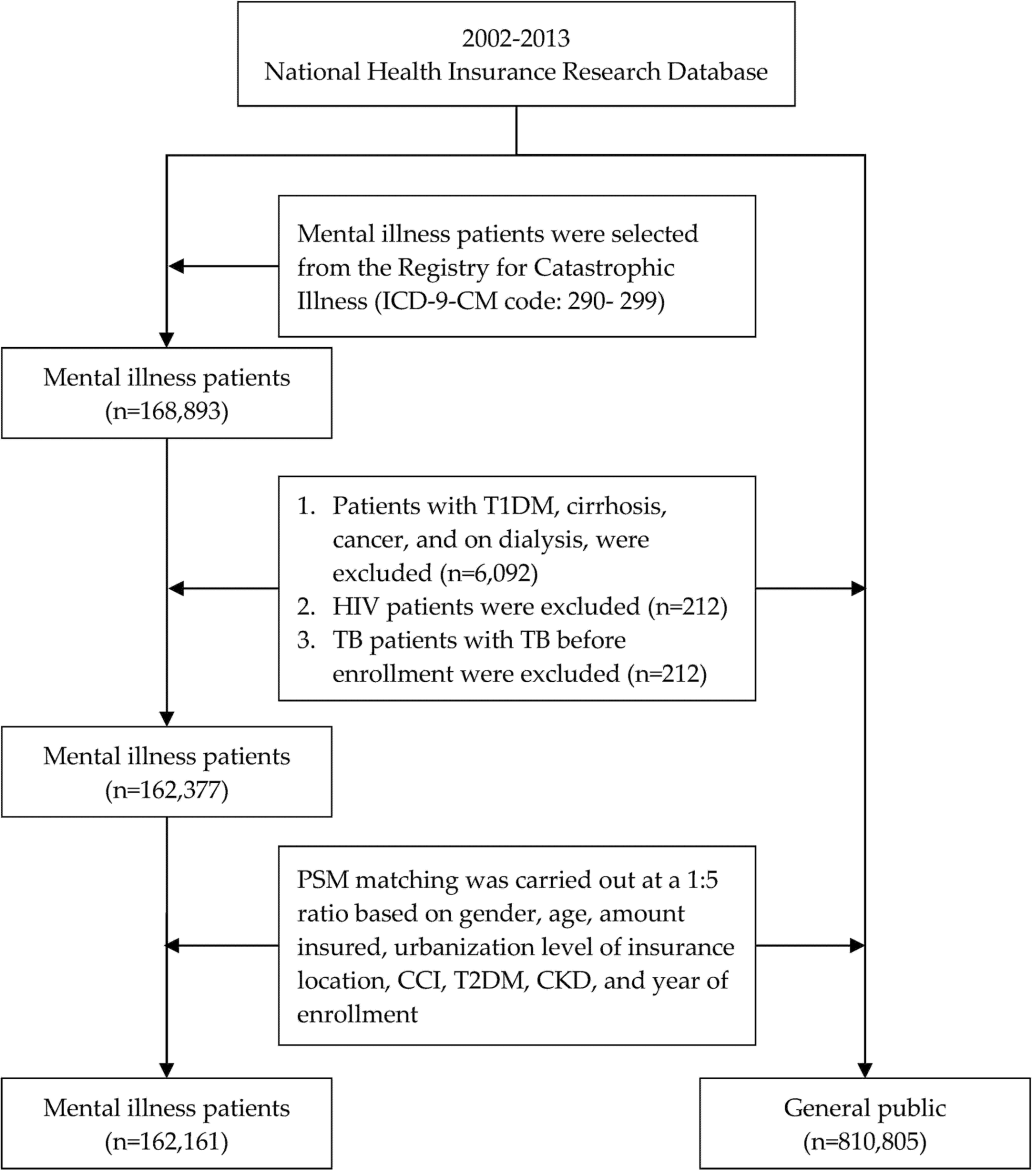
Screening process for the study subjects.

Chronic mental illness in this study is defined as persistent psychiatric disorders, including schizophrenia, bipolar disorder, schizoaffective disorder, and major depressive disorder, classified under ICD‐9‐CM Codes 290–299. Patients diagnosed between 2002 and 2013 were identified exclusively from the Catastrophic Illness Registry within the NHIRD, which requires rigorous clinical verification to minimize overdiagnosis. This approach ensures that only individuals with severe and persistent mental disorders are included as study subjects. The prevalence of TB in mental illness patients and the general public from January 1, 2002, to December 31, 2013, was then compared, totaling 168 893 subjects. Patients with type 1 diabetes mellitus, cirrhosis, cancer, on dialysis (*n* = 6092), HIV (*n* = 212), past TB (*n* = 212), and those with an amended diagnosis were excluded. Thus, a total of 162 377 subjects were included in this study. Figure 1 illustrates the screening procedure of the subjects.

### 2.3 Definition of Variables

The definition of TB development was a TB case that was registered in the Tuberculosis Database from 2002 to 2013. A confirmed TB diagnosis in this database must conform to abnormal X‐ray diagnosis results, initial differential diagnosis review, and sputum culture result positive for *Mycobacterium tuberculosis* . Amended diagnoses were removed. Patient characteristics included sex, age, urbanization level of residence location, monthly income, Charlson comorbidity index (CCI), presence/absence of type 2 diabetes mellitus (T2DM), and presence/absence of CKD. Age was the index year when the study subject was diagnosed with a major mental illness, which was divided into five groups: < 20, 20–34, 35–49, 50–64, and ≥ 65 years. Monthly income was divided into five groups: ≤ NTD 17 880/month, NTD 17 881–22 800/month, NTD 22 801–36 300/month, NTD 36 301–45 800/month, and ≥ NTD 45 800/month. The urbanization level of residence location was divided into seven grades, wherein Grade 1 was attributed to highly urbanized towns, Grade 2 for moderately urbanized towns, Grade 3 for emerging towns, Grade 4 for normal township urban districts, Grade 5 for aged towns, Grade 6 for rural towns, and Grade 7 for remote towns [21]. T2DM (ICD‐9CM: 250, excluding 250.x1 or 250.x3) and CKD (ICD‐9CM: 585) were also noted as high‐risk diseases for developing TB in this study. Medical records of the first 2 years after the index day (when the subject was diagnosed with major mental illness) were examined, and the primary and secondary diagnosis codes resulting in three outpatient visits or one hospitalization in 1 year were used for identification. Disease severity markers developed by Deyo et al. [22] were adopted for the CCI. The CCI score of the disease condition in the first 2 years after the subject was diagnosed with a major mental illness was used for calculation and divided into four grades: 0, 1, 2, and ≥ 3 points.

### 2.4 Statistical Analysis

The statistical suite SAS 9.4 (SAS Institute Inc., Cary, NC, United States) was used in this study as an analysis tool. To address potential confounding in examining the association between mental illness and TB, we utilized propensity score matching (PSM) to construct a balanced cohort. Propensity scores, representing the conditional probability of having a mental illness given observed covariates, were estimated using a logistic regression model. The model incorporated key baseline characteristics, including sex, age, monthly salary, urbanization level, CCI, and year of inclusion. Mental illness patients were matched to individuals from the general public at a 1:5 ratio with a caliper width of 0.2 standard deviations of the logit of the propensity score to ensure precise matches. Specifically, among the 162 377 subjects in this study, 162 161 completed PSM matching, and only 216 subjects were not matched, resulting in a matching rate of 99.8%. After matching, the chi‐square test was used to analyze differences in various variables between mental illness patients and the general public.

Incidence per 100 000 person‐years was used as a marker for the description and comparison of TB incidence between the general public and mental illness patients. Regarding the comparison of the risk of developing the disease, the log‐rank test was first performed, and bivariate analysis was carried out for the risk of developing TB in both patients. Afterward, the conditional Cox proportional hazards model was used to control related variables before analyzing the relative risks of developing TB in both patients. The adjustment variables contained sex, age, monthly salary, urbanization, CCI, and related comorbidities. Finally, the cumulative risk curves of developing TB for the general public and mental illness patients were plotted.

## 3 Results

Table 1 shows the baseline characteristics of 162 161 mental illness patients and 810 805 matched controls (1:5 ratio) from 2002 to 2013. No significant differences were observed in sex, age, monthly salary, urbanization level, CCI, T2DM, or CKD (all *p* > 0.05), confirming effective propensity score matching.

**TABLE 1:** Distribution of basic characteristics of mental illness patients and normal patients after matching.

Variable	Total		General public		Mental illness patients		*p* value
	*n*	%	*n*	%	*n*	%	
Total	972 966	100.00	810 805	100.00	162 161	100.00	
Sex							0.974
Female	513 414	52.77	427 851	52.77	85 563	52.76	
Male	459 552	47.23	382 954	47.23	76 598	47.24	
Age, years							1.000
< 20	60 379	6.21	50 304	6.20	10 075	6.21	
20–34	210 859	21.67	175 719	21.67	35 140	21.67	
35–49	243 737	25.05	203 137	25.05	40 600	25.04	
50–64	171 153	17.59	142 616	17.59	28 537	17.60	
≥ 65	286 838	29.48	239 029	29.48	47 809	29.48	
Mean ± SD	49.20 ± 20.93		49.02 ± 20.67		50.09 ± 22.16		
Monthly salary (NTD)							1.000
≤ 17 880	366 095	37.63	305 079	37.63	61 016	37.63	
17 881–22 800	369 060	37.93	307 559	37.93	61 501	37.93	
22 801–36 300	110 171	11.32	91 807	11.32	18 364	11.32	
36 301–45 800	55 347	5.69	46 125	5.69	9222	5.69	
≥ 45 801	72 293	7.43	60 235	7.43	12 058	7.44	
Urbanization							1.000
Level 1	244 057	25.08	203 390	25.08	40 667	25.08	
Level 2	310 547	31.92	258 809	31.92	51 738	31.91	
Level 3	159 595	16.40	133 005	16.40	26 590	16.40	
Level 4	144 336	14.83	120 291	14.84	24 045	14.83	
Level 5	22 697	2.33	18 901	2.33	3796	2.34	
Level 6	47 527	4.88	39 568	4.88	7959	4.91	
Level 7	44 207	4.54	36 841	4.54	7366	4.54	
CCI^a^							1.000
0	418 382	43.00	348 655	43.00	69 727	43.00	
1	204 455	21.01	170 367	21.01	34 088	21.02	
2	123 665	12.71	103 051	12.71	20 614	12.71	
≥ 3	226 464	23.28	188 732	23.28	37 732	23.27	
Type 2 diabetes mellitus							0.655
No	847 885	87.14	706 626	87.15	141 259	87.11	
Yes	125 081	12.86	104 179	12.85	20 902	12.89	
Chronic kidney disease							0.869
No	957 944	98.46	798 294	98.46	159 650	98.45	
Yes	15 022	1.54	12 511	1.54	2511	1.55	

^a^ CCI calculation does not include type 2 diabetes mellitus.

Table 2 presents TB incidence per 100 000 person‐years. Mental illness patients had a higher TB incidence (87) than controls (71). By sex, TB incidence was elevated in both male (120 vs. 103) and female (59 vs. 42) mental illness patients compared to controls. By age, mental illness patients aged < 20 years showed slightly lower TB incidence (7 vs. 8), but all other age groups had higher incidence than controls, notably those ≥ 65 years (278 vs. 195). For monthly salary, TB incidence was comparable at NTD 36 301–45 800 (53 vs. 53) but higher in mental illness patients across other income ranges. By urbanization, mental illness patients in Grade 6 rural towns had lower TB incidence (91 vs. 130), whereas other levels showed higher incidence than controls. Across all CCI scores, TB incidence was consistently higher in mental illness patients. For comorbidities, mental illness patients with or without T2DM (206 vs. 157; 74 vs. 60) or CKD (243 vs. 237; 86 vs. 69) had higher TB incidence than controls, with incidence ratios ranging from 1.02 to 1.31.

**TABLE 2:** Incidence of tuberculosis in mental illness patients and normal patients per 100 000 person‐years.

Variable	Mental illness patients		General public	
	No. of people who developed tuberculosis	Per 100 000 person years incidence	No. of people who developed tuberculosis	Per 100 000 person years incidence
Total	883	87	3935	71
Sex				
Female	316	59	1230	42
Male	567	120	2705	103
Age, years				
< 20	5	7	29	8
20–34	45	18	189	14
35–49	108	38	443	30
50–64	117	62	498	50
≥ 65	608	278	2776	195
Monthly salary (NTD)				
≤ 17 880	338	87	1446	69
17 881–22 800	380	100	1776	84
22 801–36 300	71	62	265	43
36 301–45 800	31	53	167	53
≥ 45 801	63	86	281	69
Urbanization				
Level 1	188	73	773	55
Level 2	231	70	1037	58
Level 3	158	95	568	63
Level 4	138	94	717	87
Level 5	41	180	179	140
Level 6	42	91	339	130
Level 7	85	195	322	129
CCI^a^				
0	128	26	605	24
1	180	80	660	54
2	180	146	745	105
≥ 3	395	219	1925	169
Type 2 diabetes mellitus				
No	677	74	2965	60
Yes	206	206	970	157
Chronic kidney disease				
No	863	86	3801	69
Yes	20	243	134	237

^a^ CCI calculation does not include type 2 diabetes mellitus.

**FIGURE 2:**
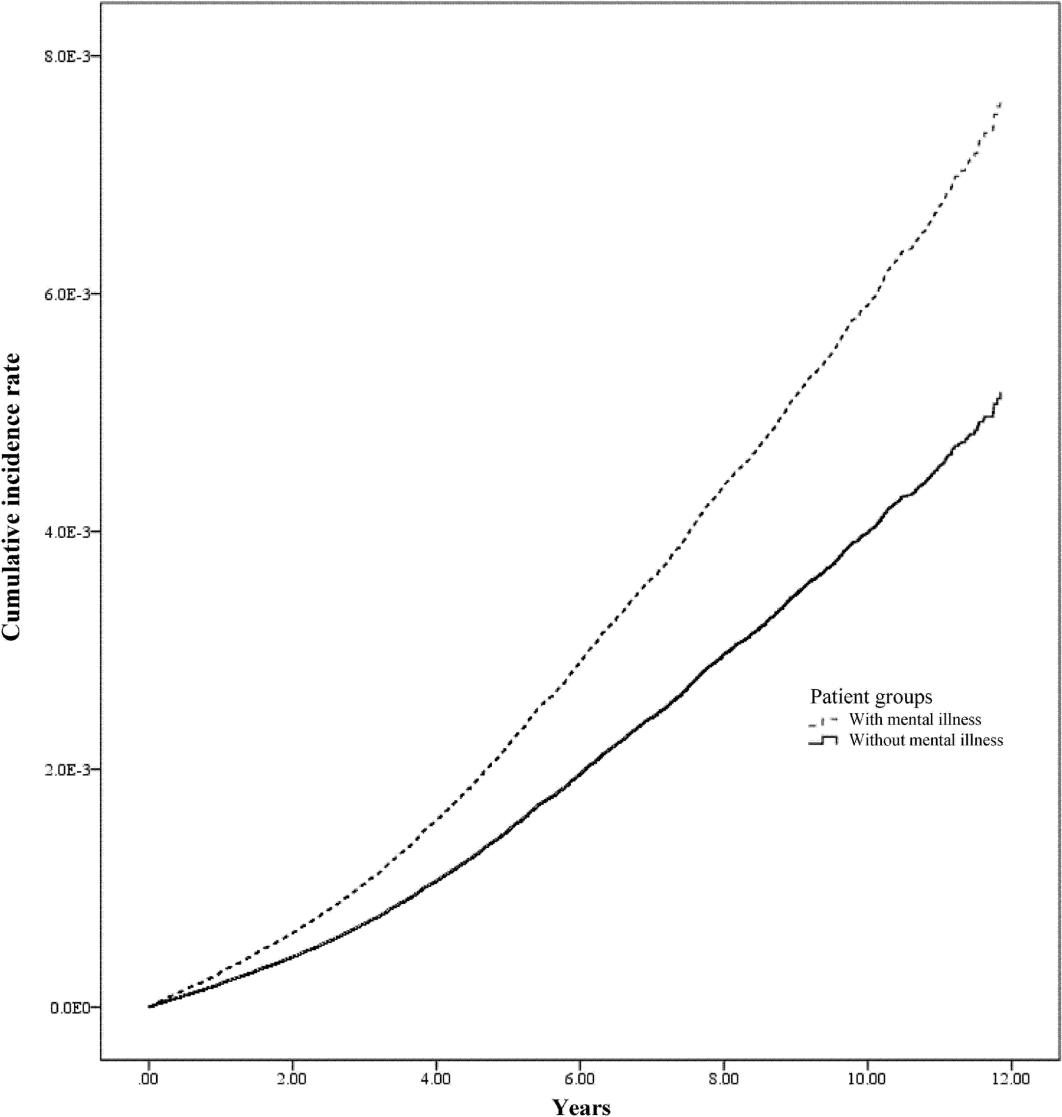
The cumulative incidence rate of the risk of developing tuberculosis between mental illness patients and the general public (log rank test < 0.001).

Table 3 summarizes TB risk. After confounding variables were adjusted for, the adjusted conditional Cox proportional hazards model confirmed a 1.48‐fold higher TB risk in mental illness patients (95% CI: 1.38–1.59). Figure 2 illustrates the cumulative TB incidence, showing a significantly higher rate in mental illness patients compared to the general public. In addition, TB risk increased with age (HR ranging from 1.86 for 20–34 years to 21.82 for ≥ 65 years relative to < 20 years) and decreased with higher monthly salary (HR ranging from 0.97 for NTD 17 881–22 800 to 0.78 for NTD 36 301–45 800 relative to ≤ 17 880). Patients with T2DM had a higher risk of TB (HR: 1.22, 95% CI: 1.13–1.31), a trend similarly observed among patients with CKD (HR: 1.24, 95% CI: 1.06–1.47).

**TABLE 3:** Relative risk of developing tuberculosis between mental illness patients and normal patients.

Variable	Unadjusted model			Adjusted model		
	HR	95% CI	*p* value	HR	95% CI	*p* value
Mental illness						
No (ref.)	1			1		
Yes	1.25	1.16–1.34	< 0.001	1.48	1.38–1.59	< 0.001
Sex						
Female (ref.)	1			1		
Male	2.38	2.24–2.53	< 0.001	2.98	2.80–3.16	< 0.001
Age, years						
< 20 (ref.)	1			1		
20–34	1.82	1.27–2.61	0.001	1.86	1.30–2.67	< 0.001
35–49	3.79	2.68–5.36	< 0.001	3.89	2.75–5.51	< 0.001
50–64	6.39	4.52–9.02	< 0.001	6.38	4.51–9.04	< 0.001
≥ 65	26.57	18.95–37.24	< 0.001	21.82	15.44–30.82	< 0.001
Monthly salary (NTD)						
≤ 17 880 (ref.)	1			1		
17 881–22 800	1.20	1.13–1.28	< 0.001	0.97	0.91–1.04	0.415
22 801–36 300	0.65	0.58–0.73	< 0.001	0.84	0.74–0.94	0.003
36 301–45 800	0.74	0.64–0.85	< 0.001	0.78	0.68–0.91	0.001
≥ 45 801	1.01	0.90–1.13	0.897	0.85	0.75–0.95	0.005
Urbanization						
Level 1 (ref.)	1			1		
Level 2	1.03	0.95–1.12	0.463	1.03	0.95–1.12	0.485
Level 3	1.17	1.06–1.29	0.001	1.16	1.06–1.28	0.002
Level 4	1.53	1.39–1.68	< 0.001	1.28	1.16–1.41	< 0.001
Level 5	2.53	2.18–2.93	< 0.001	1.83	1.57–2.13	< 0.001
Level 6	2.17	1.93–2.44	< 0.001	1.54	1.36–1.74	< 0.001
Level 7	2.40	2.14–2.70	< 0.001	1.88	1.67–2.13	< 0.001
CCI^a^						
0 (ref.)	1			1		
1	2.40	2.17–2.65	< 0.001	1.26	1.13–1.40	< 0.001
2	4.58	4.16–5.05	< 0.001	1.50	1.34–1.67	< 0.001
≥ 3	7.44	6.85–8.09	< 0.001	1.66	1.49–1.84	< 0.001
Type 2 diabetes mellitus						
No (ref.)	1			1		
Yes	2.74	2.57–2.93	< 0.001	1.22	1.13–1.31	< 0.001
Chronic kidney disease						
No (ref.)	1			1		
Yes	3.60	3.07–4.23	< 0.001	1.24	1.06–1.47	0.009

^a^ CCI calculation does not include type 2 diabetes mellitus.

## 4 Discussion

The study results indicated that TB incidence was higher among patients with mental illness compared to the general population. After adjusting for relevant variables, the risk of developing TB in this group was 1.48 times that of the general public, which was similar to that of previous studies [2, 6, 15, 16].

The increased risk of TB in patients with chronic mental illness is a multifaceted issue overlapping social, behavioral, and biological factors [23, 24, 25, 26, 27]. Biologically, chronic mental disorders, such as depression and schizophrenia, can dysregulate immune function, increasing susceptibility to infections like TB. Studies have reported hazard ratios for TB ranging from 1.15 to 2.63 for depression and from 1.52 to 3.04 for schizophrenia, potentially due to impaired T‐cell responses and elevated proinflammatory cytokines that compromise the body's ability to control *M. tuberculosis* [28]. Socioeconomically, patients with chronic mental illness often face poverty, malnutrition, and limited healthcare access, which are established TB risk factors. Consistent with this, our study found that the TB risk decreased with a higher monthly salary. Behaviorally, poor illness insight frequently leads to nonadherence to medical care, delaying TB diagnosis and treatment [23, 24, 25, 26, 27]. Environmentally, overcrowding in mental health facilities, often characterized by poor ventilation, facilitates airborne TB transmission, as evidenced by studies linking higher patient density per room to increased active TB cases [9, 12, 29]. These factors collectively create a blind spot in TB prevention, underscoring the need for integrated mental health and TB control strategies.

Our study findings indicate that TB risk increases with age, highlighting age as a significant determinant of TB susceptibility. Specifically, the adjusted hazard ratios ranged from 1.86 for the 20–34 age group to 21.82 for those aged ≥ 65 years compared to individuals < 20 years (Table 3). This trend is primarily driven by age‐related immune decline, often termed immunosenescence, which impairs T‐cell‐mediated responses critical for controlling *M. tuberculosis* infection. Additionally, the reactivation of latent TB infection becomes more likely with advancing age. These factors collectively elevate TB incidence in older populations, as supported by studies identifying immunosenescence and latent infection reactivation as key drivers of TB risk in the elderly [30, 31]. Our study also found that patients with T2DM or CKD had a higher risk of TB, compared to those without these comorbidities. In T2DM, poor glycemic control and impaired innate immunity, such as reduced macrophage phagocytic activity, contribute to increased TB susceptibility [32, 33, 34]. For CKD, uremia, malnutrition, and altered cellular immunity, including T‐cell dysfunction, elevate the risk of TB, particularly through reactivation of latent infections [35, 36].

This study is a nationwide population‐based study in Taiwan. The generalizability of our findings to other populations should be considered in the healthcare system and socioeconomic environment. These associations are likely applicable to countries with similar universal healthcare systems and high TB prevalence. However, in low‐TB‐incidence countries, the magnitude of risk may differ due to variations in TB exposure, diagnostic capacity, and mental health care access. Thus, local TB epidemiology and healthcare infrastructure must be considered when applying these results to other populations.

Despite the strengths of our study, several limitations may introduce potential biases. First, reliance on the NHIRD and Taiwan's Tuberculosis Database limits the inclusion of key variables, such as smoking, alcohol consumption, drug use, residence environment, contact history, and latent tuberculosis infection (LTBI) status, a critical target for TB management. The absence of these data may lead to unmeasured confounding, potentially overestimating the TB risk associated with chronic mental illness, T2DM, or CKD. Second, the retrospective cohort design restricts our ability to establish causality, as unmeasured confounders (e.g., substance use and incarceration history) could inflate observed associations. Third, our study only included mental illness patients registered in the Catastrophic Illness Registry, which requires rigorous clinical verification, excluding milder cases and limiting generalizability to all chronic mental illness populations. This selection bias may overestimate TB risk by focusing on severe cases. Fourth, potential misclassification or underreporting in claims data, particularly for mental disorders or TB diagnoses, could lead to misclassification bias, either underestimating or overestimating risk estimates. Finally, the exclusion of high‐risk subgroups (e.g., patients with HIV, dialysis, or prior TB) further limits applicability to these populations.

Our findings underscore critical implications for clinical practice and public health in managing TB among patients with mental illness. Clinically, healthcare providers should integrate routine TB screening into the care of patients with chronic mental illness. Enhanced monitoring for TB symptoms in mental health, where overcrowding and delayed diagnosis are concerns, is essential. From a public health perspective, these findings advocate for tailored TB prevention programs, including prophylactic treatment for latent TB infection in these populations and improved infection control measures in high‐risk settings.

## 5 Conclusion

The risk of developing TB in mental illness was higher than that of the general public, and the difference in this risk between the two increased with time. Although the link between mental illness and increased TB risk is well established, the precise causal mechanisms remain unclear. Nevertheless, TB screening in mental illness patients will help in the identification of potential TB cases, and early awareness will help prevent the disease from spreading.

## Author Contributions

**Li‐Chen Hung:** conceptualization, data curation, formal analysis, methodology, validation, writing – original draft, writing – review and editing. **Wen‐Chen Tsai:** conceptualization, data curation, funding acquisition, methodology, writing – review and editing. **Kuang‐Hua Huang:** conceptualization, funding acquisition, methodology, validation, writing – review and editing. **Pei‐Tseng Kung:** funding acquisition, methodology, validation, writing – review and editing. **Tung‐Han Tsai:** formal analysis, writing – review and editing.

## Ethics Statement

This study was performed in accordance with the Declaration of Helsinki. This study protocol was approved by China Medical University and Hospital Research Ethics Committee—Approval No. CRREC‐109‐100. Participant consent was not required because this was a retrospective study using deidentified data; the need for informed consent was waived.

## Conflicts of Interest

The authors declare no conflicts of interest.

## Data Availability

This study used databases including the National Health Insurance Research Database, Registry for Catastrophic Illness Patients, Tuberculosis Database, and Household Registration Records of Taiwan. Data were published and managed by the Ministry of Health and Welfare, Taiwan. Due to legal restrictions imposed by the Taiwan government related to the Personal Information Protection Act, these databases cannot be made publicly available. All researchers can apply for using the databases for conducting their studies. Requests for using data can be sent as a formal proposal to the Science Center of the Ministry of Health and Welfare (https://www.mohw.gov.tw/mp‐2.html). Any raw data are not allowed to be brought out from the Science Center. Only the analytic outputs in format of table or figure can be printed out. The restrictions prohibited the authors from making the minimal data set publicly available.
